# Impact of the systematic introduction of tomosynthesis on breast biopsies: 10 years of results

**DOI:** 10.1007/s11547-023-01640-7

**Published:** 2023-05-17

**Authors:** Daniele La Forgia, Rahel Signorile, Samantha Bove, Francesca Arezzo, Gennaro Cormio, Antonella Daniele, Miriam Dellino, Annarita Fanizzi, Gianluca Gatta, Miria Lafranceschina, Raffaella Massafra, Alessandro Rizzo, Francesco Alfredo Zito, Emanuele Neri, Lorenzo Faggioni

**Affiliations:** 1 Istituto Tumori Giovanni Paolo II, I.R.C.C.S, Via Orazio Flacco 65, 70124 Bari, Italy; 2 Department of Interdisciplinary Medicine (DIM), University of Bari Aldo Moro, 70121 Bari, Italy; 3 Clinic of Obstetrics and Gynecology, San Paolo Hospital, 70123 Bari, Italy; 4 Department of Biomedical Sciences and Human Oncology, University of Bari Aldo Moro, 70100 Bari, Italy; 5 Breast Unit, Department of Clinical and Experimental Internship, University of Campania Luigi Vanvitelli, Via De Crecchio 7, 80138 Naples, Italy; 6 Academic Radiology, Department of Translational Research, University of Pisa, Via Roma 67, 56126 Pisa, Italy

**Keywords:** Breast neoplasm, Full-field digital mammography, Digital breast tomosynthesis, Breast core biopsy, VABB

## Abstract

Digital Breast Tomosynthesis (DBT) is a cutting-edge technology introduced in recent years as an in-depth analysis of breast cancer diagnostics. Compared with 2D Full-Field Digital Mammography, DBT has demonstrated greater sensitivity and specificity in detecting breast tumors. This work aims to quantitatively evaluate the impact of the systematic introduction of DBT in terms of Biopsy Rate and Positive Predictive Values for the number of biopsies performed (PPV-3). For this purpose, we collected 69,384 mammograms and 7894 biopsies, of which 6484 were Core Biopsies and 1410 were stereotactic Vacuum-assisted Breast Biopsies (VABBs), performed on female patients afferent to the Breast Unit of the Istituto Tumori “Giovanni Paolo II” of Bari from 2012 to 2021, thus, in the period before, during and after the systematic introduction of DBT. Linear regression analysis was then implemented to investigate how the Biopsy Rate had changed over the 10 year screening. The next step was to focus on VABBs, which were generally performed during in-depth examinations of mammogram detected lesions. Finally, three radiologists from the institute’s Breast Unit underwent a comparative study to ascertain their performances in terms of breast cancer detection rates before and after the introduction of DBT. As a result, it was demonstrated that both the overall Biopsy Rate and the VABBs Biopsy Rate significantly decreased following the introduction of DBT, with the diagnosis of an equal number of tumors. Besides, no statistically significant differences were observed among the three operators evaluated. In conclusion, this work highlights how the systematic introduction of DBT has significantly impacted the breast cancer diagnostic procedure, by improving the diagnostic quality and thereby reducing needless biopsies, resulting in a consequent reduction in costs.

## 1. Introduction

The American Cancer Society (ACS) [40] estimates that cancer represents the second major cause of death in the USA and a primary health concern worldwide. Findings published by the ACS in 2022 show that breast cancer incidence in the USA has risen, that is to say, there was a 0.5% annual increase between 2010 and 2019, whereas mortality rates have declined [17]. Although mammography screening programs have contributed to reducing breast cancer mortality by up to 30%, by encouraging early diagnosis [42], the mammogram’s sensitivity is significantly reduced in the case of dense breasts, which are breasts characterized by a higher component of fibro-glandular matter which require closer follow-up and the employment of further methods, such as manual or automated ultrasound [14, 19, 30]. Currently, the presence of dense tissue is the main reason for failed early diagnosis in mammography screening programs, increasing the likelihood of a diagnosis of advanced breast cancer [8]. Over the years, the issue of dense breasts has gained more importance in the scientific community since legislation was introduced in the USA to assess both the density levels and the risks associated with reduced mammographic sensitivity [25, 27]: Differently from dense breasts, the non-dense ones have recently been associated with increased cardiovascular risk [37].

Moreover, in recent years, several cutting-edge techniques have been developed to overcome problems associated with the sensitivity of 2D FFDM (Full-Field Digital Mammography). An example is DBT (Digital Breast Tomosynthesis), which generates three-dimensional breast images through a series of acquisitions at different angles, which partly solves the masking effect linked to the tissue density and increases both the sensitivity and specificity of the investigation [16, 22, 33]. Specifically, DBT can highlight small masses and distortions better than 2D FFDM [44], though there seems to be no significant improvement in detecting microcalcifications [26].

The US Food and Drug Administration originally approved the use of DBT equipment for screening, but only in combination with 2D mammography (FFDM or synthesized) [12, 29]. Nonetheless, the combined use of DBT and 2D FFDM would significantly increase the amount of radiation exposure per examination and the execution time compared with the implementation of the 2D FFDM screening alone. Furthermore, current DBT protocols involve radiation doses comparable to those used in 2D FFDM, depending on the equipment used, the imaging protocol, and the breast thickness [13, 15, 26, 31, 32]. Therefore, to improve the diagnostic accuracy without significantly increasing the patients’ radiation exposure, several centers, including the Breast Unit of the Istituto Tumori “Giovanni Paolo II” of Bari, decided on the exclusive and systematic use of the DBT.

Thus far, several state-of-the-art retrospective studies have focused on comparing FFDM and DBT performances in terms of either sensitivity or specificity or both [18]. Furthermore, in many studies, the performances of radiologists in breast cancer screening were compared both before and after the systematic introduction of DBT in terms of recall rate and cancer detection rate [41]. Finally, Sharma et al. in their work [39] investigated variations in the benign biopsy rate (the number of benign tumors diagnosed out of the total number of biopsies performed).

Nonetheless, to the best of our knowledge, the B5 Biopsy Rate, that is, the number of malignant tumors diagnosed out of the total number of biopsies performed, along with Positive Predictive Values for biopsies performed (PPV-3), that is the ratio between the detected cancers and the number of biopsies performed [22], has yet to be investigated in order to compare the performances of FFDM and DBT.

The aim of this work is, therefore, to evaluate the variations of both the Biopsy Rate and the PPV-3 over a 10 year period of diagnostic activity at a Breast Unit of an Italian Oncological Institute regarding the exclusive use of FFDM and the exclusive use of DBT to verify if the systematic introduction of DBT produced any improvement in terms of better diagnostic accuracy and reduction in needless biopsy samples.

## Materials and methods

### Experimental data

For this retrospective study (approved by the Scientific Board of the Istituto Tumori "Giovanni Paolo II” of Bari, Italy), 69,384 consecutive mammograms of women participants were collected from January 1, 2012, to December 31, 2021, and clinical diagnostic mastology checkups were carried out from Monday to Friday, by 2 teams, each consisting of 1 radiologist, 1 nurse and 1 radiology technician dedicated to first-level diagnostics on a total of 40 patients (20 per team) divided as follows: first access (*n* = 6), patients with oncological family history (*n* = 6), controls (*n* = 5), oncological patients in follow-up (*n* = 17), and emergencies (*n* = 6). It follows that 6/40 were symptomatic patients, while 34/40 were asymptomatic (on the day): Among the latter, there were 17 cancer patients who had already undergone surgery in follow-up, 6 patients with oncological family history linked to breast cancer and 11 with no particular risks. All mammograms were acquired and analyzed by radiologists with an experience of over 15 years and afferent to the Breast Unit of our institute. More specifically, FFDMs were acquired from 2012 to 2015, whereas DBTs were acquired from 2017 to 2021. Meanwhile, a combined acquirement of FFDMs and DBT was performed in 2016, before the exclusive and systematic introduction of the DBT. In addition, we collected data on the number of mammograms performed, the total number of biopsies required, the estimated BIRADS, the average patient’s age, and the bioptic histological class, the results of which were classified from B1 to B5, according to European Guidelines and supplements [7, 34, 35]: B1, normal tissue; B2, benign abnormalities; B3, a heterogeneous group of lesions of unknown biologic potential; B4, suspicious findings but insufficient for a definite diagnosis of malignancy; and B5, unequivocal malignancy.

### Characteristics of radiological techniques

DBT involves multiple projections acquired across an arc which are reconstructed into a series of stacked images [5]. Depending on the manufacturer, differently from FFDM, during DBT image acquisition, the x-ray tube pivots in an arc that varies between 15° (narrow range) and 60° (wide range) in a plane aligned with the chest wall. In general, the wider angular range of X-ray tube motion produces more tomographic information and enables better section separation or better vertical resolution (z-axis). Increasing the angular range for the tube movement requires more projections for sufficient sampling [5].

Mammography investigations were conducted on the same device throughout the observation period (GE Healthcare Senographe Essential™) before and after the introduction of the tomosynthesis module. During the period of the introduction of tomosynthesis (2016–2021), investigations were performed by the same dedicated radiologists.

On average, the radiation dose was about 30% higher in the DBT plus SM (Synthesized Mammography) protocol compared to the FFDM data, which was however, considered acceptable and in line with the European guidelines for quality assurance in mammography screening [1, 3, 24, 28, 34].

### Statistical analysis

For each year from 2012 to 2021, we evaluated both the Biopsy Rate and PPV-3. Specifically, we focused on B5 tumors. Therefore, to evaluate how the Biopsy Rate changed over the 10 year period, we performed a linear regression analysis, considering the estimated linear association as statistically significant when the *p*-value resulted as less than 0.05. Furthermore, we investigated whether or not there was any significant interobserver variability in detecting neoplasms, by comparing the performances of three different operators by means of the Wilcoxon statistical test. All analyses were performed using RStudio 2022.02.3 statistical software.

## Results

According to the data reported in the Materials and Methods section, the examined population consisted of 85% asymptomatic patients and 15% symptomatic patients. More specifically, 42.5% of the patients were on oncological follow-up, 15% of the patients presented with familiar risk and 27.5% of the patients with no significant risk at all.

The first analysis evaluated how the overall Biopsy Rate had changed over the 10 year period. For this purpose, Table 1 shows the number of mammograms performed, the overall number of biopsies required, the number of detected B5 tumors, in addition to the estimated Biopsy Rate and PPV-3 for each year from 2012 to 2021.

**Table 1:** Detailed overview of the total mammograms performed, biopsies required, and detected B5 tumors from 2012 to 2021. For each year, the Biopsy Rate and the PPV-3 are also reported

Year	Mammograms	Biopsies	B5 tumors	Biopsy rate (%)	PPV-3 (%)
2012	3247	594	278	18.3	46.8
2013	2731	462	254	16.9	55.0
2014	5665	576	343	10.2	59.5
2015	6968	642	350	9.2	54.5
2016	7373	927	327	12.6	35.3
2017	7314	828	376	11.3	45.4
2018	8556	1001	435	11.7	43.5
2019	9751	1034	436	10.6	42.2
2020	8497	866	338	10.2	39.0
2021	9282	965	357	10.4	37.0

**Fig. 1:**
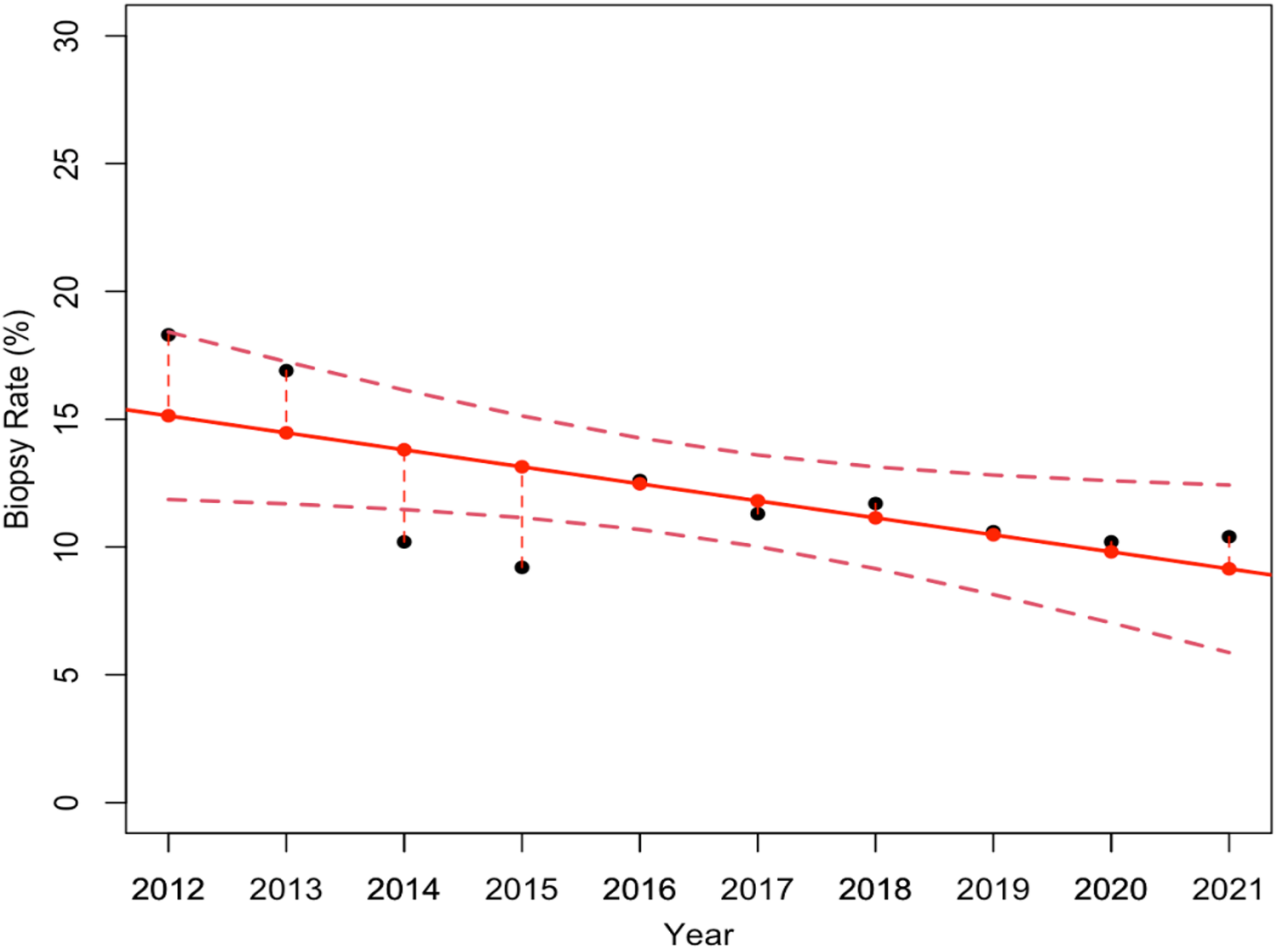
Linear regression model, which estimates how the overall Biopsy Rate changed over the 10 year screening. The model resulted statistically significant with a *p*-value equal to 0.04

Thus, starting from the data collected in Table 1, we estimated the first linear regression model (Fig. 1), which describes the overall Biopsy Rate variations over the 10 year period. Specifically, Fig. 1 shows an overall reduction in biopsies carried out during this period. The statistical test confirmed that the estimated model was statistically significant, with a *p*-value of 0.04.

**Fig. 2:**
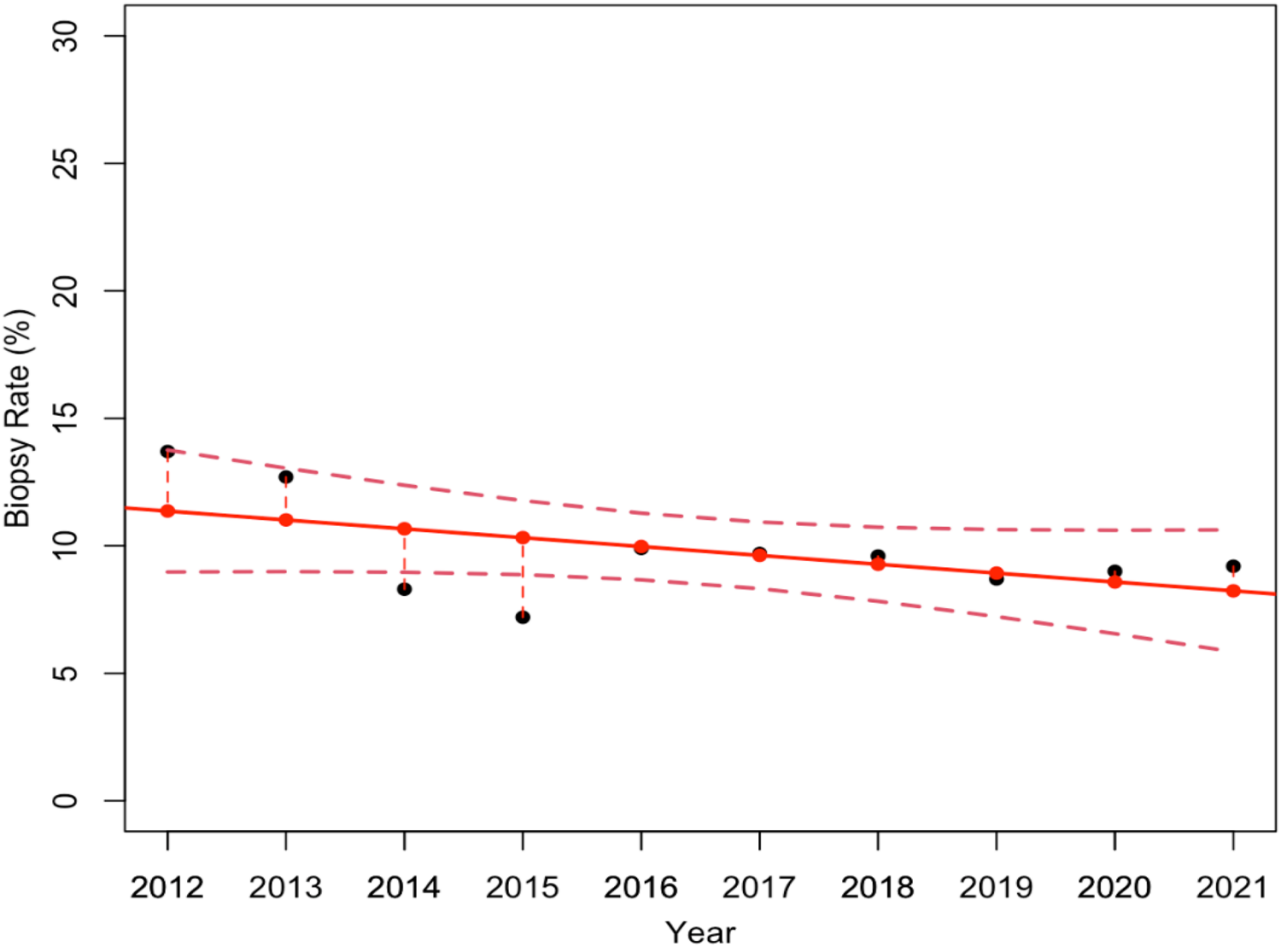
Linear regression model estimates how the Biopsy Rate related to CBs changed over the 10 year period. The model resulted not statistically significant, with a *p*-value equal to 0.11

**Fig. 3:**
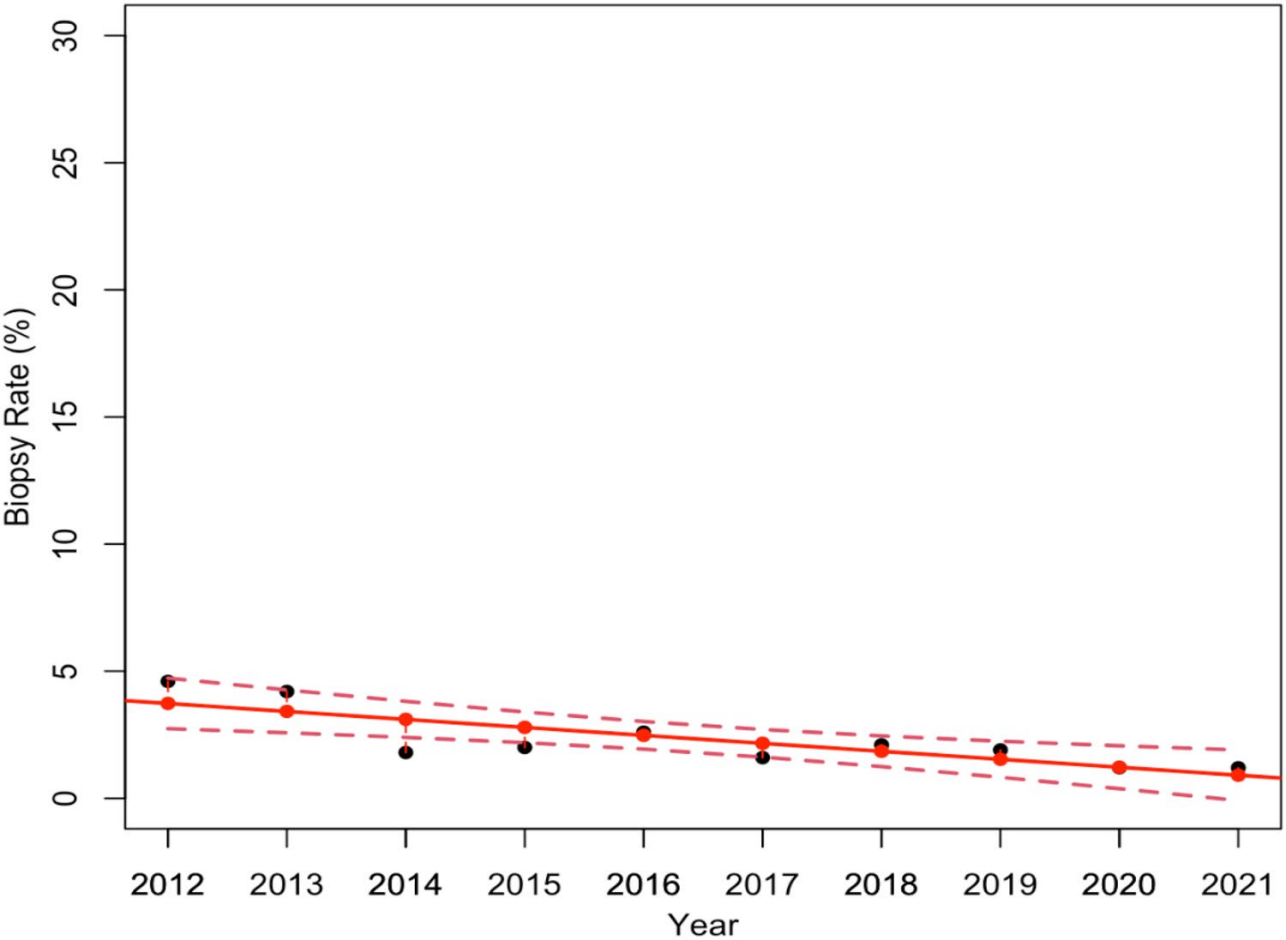
Linear regression model estimates how the Biopsy Rate related to VABBs changed over the 10 year period. The model resulted statistically significant with a *p*-value less than 0.005

The overall Biopsy Rate investigated so far considers both CBs and VABBs. Nonetheless, since VABBs are the biopsies generally used for in-depth analysis of mammogram detected lesions, our second goal was to independently investigate both the Biopsy Rate and the PPV-3 related to the CBs and VABBs. Thus, Tables 2 and 3 reported the number of mammograms performed, the individual number of biopsies required, the number of detected B5 tumors, in addition to the estimated Biopsy Rate and PPV-3, for each individual year from 2012 to 2021, for CBs and VABBs, respectively. To demonstrate that the two samples considered were homogeneous, we computed the average age of the patients who underwent the two different procedures beforehand: the average age was 54 years (54.20±3.52) for the patients who received VABB 55 years (55.30±1.10) for the patients who underwent CB. Consequently, from data collected in both Tables 2 and 3, we estimated two linear regression models which describe the Biopsy Rate variations over the 10 year screenings for CB (Fig. 2) and VABB (Fig. 3), respectively. As a result, even though Fig. 2 shows an overall reduction in CBs required over the years, the associated linear regression model proved not to be statistically significant with a *p*-value equal to 0.11. On the other hand, Fig. 3 shows a relevant reduction of VABBs performed during this period, particularly after the systematic introduction of DBT in 2017, confirming that the estimated model was statistically significant with a *p*-value of less than 0.005.

**Table 2:** Detailed overview of the total number of mammograms performed, CBs required, and detected B5 tumors from 2012 to 2021. For each year, the Biopsy Rate and the PPV-3 are also reported

Year	Mammograms	CBs	B5 tumors	CB Biopsy Rate (%)	PPV-3 (%)
2012	3247	444	232	13.7	52.3
2013	2731	348	219	12.7	62.9
2014	5665	473	309	8.3	65.3
2015	6968	501	300	7.2	59.9
2016	7373	733	282	9.9	38.5
2017	7314	710	338	9.7	47.6
2018	8556	818	371	9.6	45.4
2019	9751	845	381	8.7	45.1
2020	8497	761	294	9.0	38.6
2021	9282	852	323	9.2	37.9

**Table 3:** Detailed overview of the total number of mammograms performed, stereotactic VABBs required, and detected B5 tumors from 2012 to 2021. For each year, the Biopsy Rate and the PPV-3 are also reported

Year	Mammograms	VABBs	B5 tumors	VABB Biopsy Rate (%)	PPV-3 (%)
2012	3247	150	46	4.6	30.7
2013	2731	114	35	4.2	30.7
2014	5665	103	34	1.8	33.0
2015	6968	142	50	2.0	35.2
2016	7373	194	45	2.6	23.2
2017	7314	118	38	1.6	32.2
2018	8556	182	64	2.1	35.2
2019	9751	189	55	1.9	29.1
2020	8497	105	44	1.2	41.9
2021	9282	113	34	1.2	30.1

**Fig. 4:**
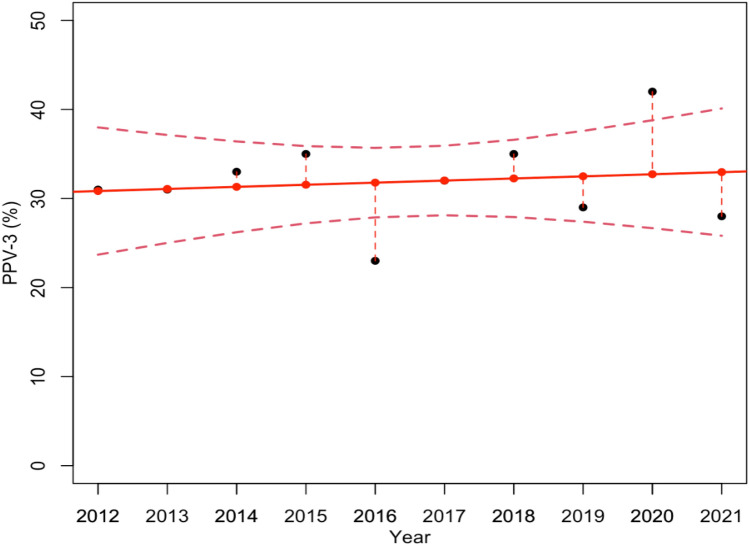
Linear regression model estimates how the PPV-3 related to VABBs changed over the 10 year screening. The model resulted not statistically significant, with a *p*-value equal to 0.69

The previously discussed results suggest that the systematic introduction of DBT in 2017 allowed an effective reduction in the number of biopsies required by radiologists, particularly with reference to VABBs. Nonetheless, this reduction could also be the consequence of a decrease in the number of B5 tumors diagnosed, leading to fewer biopsies. To investigate how the rate of B5 tumor diagnosis changed over the 10 year screening, we estimated a further linear regression model which describes the variations of the PPV-3 in relation to VABBs over time (Fig. 4). As shown in Fig. 4, the PPV-3 related to VABBs, namely, the overall percentage rate of B5 tumor diagnosed VABBs required, does not undergo significant variations over time. As a matter of fact, the estimated model proved not to be statistically significant with a *p*-value equal to 0.69, demonstrating that the decrease in the number of VABBs required was not the consequence of a reduction of B5 tumors diagnosed. Furthermore, this reduction is more evident in the data referring to VABBs, on the basis of the two periods of interest: from 2012 to 2015 with the acquisition of only FFDMs, and from 2017 to 2021 with the systematic use of DBT (Table 4).

**Table 4:** Detailed overview of the total number of mammograms performed, stereotactic VABBs required, and detected B5 tumors according to on the basis of the two periods of interest: from 2012 to 2015 and from 2017 to 2021. Both the Biopsy Rate and the PPV-3 are reported for each period

Period	Mammograms	VABBs	B5 tumors	VABBs Biopsy Rate (%)	PPV-3 (%)
2012–2015	18,611	509	115	2.7	22.6
2017–2021	43,400	707	235	1.6	33.2

Finally, with the purpose of verifying a significant association between the observed experimental results and the operators’ performances, we compared those of three operators from our institute who evaluated mammograms and performed biopsies in the last six years of the study. In particular, Table 5 reports the percentage PPV-3 value of each operator, from 2016 to 2021. The performances shown in Table 5 proved to be comparable, and the Wilcoxon statistical test demonstrated no statistically significant differences among them: Op1-Op2, *p*-value 0.69; Op1-Op3, *p*-value 0.81; Op2- Op3, *p*-value 0.94.

**Table 5:** Overview of the B5 lesions found per number of biopsies performed and percentage PPV-3 values referring to three operators of our institute from 2016 to 2021

Year	Operator 1		Operator 2		Operator 3	
	B5 lesions / biopsies	PPV – 3 (%)	B5 lesions / biopsies	PPV – 3 (%)	B5 lesions / biopsies	PPV – 3 (%)
2016	24/123	20	3/17	18	18/54	33
2017	26/76	34	6/14	43	8/31	26
2018	40/118	34	5/16	31	20/50	40
2019	35/121	29	9/26	35	11/41	27
2020	24/63	38	11/33	33	9/20	45
2021	18/49	37	5/24	21	7/38	18

## Discussion

The exclusive and systematic use of the DBT in breast cancer diagnostics improved the diagnosis accuracy, in terms of sensitivity and specificity, without significantly increasing the amount of radiation that patients undergo. Specifically, DBT has proved to be extremely sensitive as it is able to highlight small masses and distortions better than 2D FFDM, and particularly allows better detection of benign lesions from images without the need for biopsies.

Several state-of-the-art studies have already compared the FFDM with the DBT in terms of sensitivity and specificity, as well as in terms of Benign Biopsy Rate and radiologists’ performances. In this study, conversely, we compared the accuracy of the two diagnostic techniques in terms of malignant (B5) Biopsy Rate and PPV-3, particularly focusing on data related to the VABB, which is the biopsy technique generally used for in-depth analysis of mammogram detected lesions. Thus, after collecting 69,384 mammograms and 7894 biopsies performed from 2012 to 2021 on female patients afferent to the Breast Unit of the Istituto Tumori “Giovanni Paolo II” of Bari, we estimated three linear regression models which describe the overall Biopsy Rate, CBs Biopsy Rate, and VABBs Biopsy Rate variations, over the 10 year period of activity. Even though we expected the stereotactic biopsy results to be the most significant, we intentionally also reported data related to the Core Biopsies, comparing each one over the 10 year period and considering the integration of CBs which were performed on the same period.

Finally, we compared the performances of three breast radiologists at our institute to verify their individual performances in detecting neoplasms.

As a result, the first linear regression model highlighted an overall reduction of the total number of biopsies carried out during the 10 years and proved to be statistically significant with a *p*-value equal to 0.04. This result was also confirmed by the estimated linear regression model for analyzing the VABB Biopsy Rate variations, on the contrary, the experimental results show no significant reduction in the Core Biopsy Rate. Although the number of cases referring to the VABB procedure is significantly lower than that of the CBs, we feel that the result which emerged might well explain the significant reduction observed in the overall sample.

As a matter of fact, there was a relevant reduction in VABBs carried out during this period, particularly after the systematic introduction of DBT in 2017 when the VABB Biopsy Rate decreased from 2.7 to 1.6%: and the estimated model resulted statistically significant with a *p*-value of less than 0.005. Besides, we demonstrated that the decrease in the number of VABBs was not as the consequence of a reduction in B5 tumors diagnosed, by means of a further linear regression model which proved not to be statistically significant (*p*-value equal to 0.69).

Our analysis demonstrates that the systematic introduction of DBT in our institute reduced the overall number of biopsies required after the first mammographic screening without compromising the diagnostic accuracy. On the basis of this, patients avoid further unnecessary examinations and costs (related to breast cancer diagnosis) are reduced. In the light of these encouraging results, in a future study, we will go on to analyze data collected across a multicenter study to confirm the significant accuracy of DBT compared to 2D FFDM in terms of Malignant Biopsy Rate and PPV-3.

It should be emphasized that our study refers to the experience of “Giovanni Paolo II” Cancer Institute alone, which has been the regional oncological reference hub for breast pathology for the last decade. Nevertheless, our findings may not be representative of other realities.

Our results supported the literature, showing that DBT simultaneously improves breast cancer detection by reducing false positive recalls with fewer biopsies performed.

A limitation could be the radiation dose which, on average, is about 30% higher in the DBT plus SM (Synthesized Mammography) protocol compared to the FFDM, data which, however, are considered acceptable and in line with the European guidelines for quality assurance in mammography screening. Additionally, the systematic use of tomosynthesis could mean a longer reading time and decreased productivity. However, in our experience, an increase in mammographic performance was observed over time for the same number of operators, with the sole exception of the first part of the COVID period: some retrospective studies in the literature have also shown that radiologists using AI tools for the simultaneous reading of DBT or a combination of several techniques could reduce the reading time by maintaining a level that is not lower or obtaining better performances in breast cancer diagnosis [2, 6, 9–11, 36] as already documented on other neoplasms [4, 20, 21, 38, 43]. A further limitation of DBT is overdiagnosis, defined as the detection of multiple low-grade lesions and small tumors, which carries economic implications that need to be considered.

Future studies to explore breast cancer growth and access to long-term clinical follow-up data are needed to fully understand the complex question of identifying and treating small and slow-growing breast cancers associated with DBT screening [23].

## Conclusions

This work highlights the significant impact of the systematic introduction of DBT on the results of biopsy investigations, particularly stereotaxic: the technique improves overall sensitivity and specificity in diagnosing malignant neoplastic pathology in line with what is documented in the literature. The greater specificity would also significantly reduce the number of unnecessary stereotaxic biopsy investigations with a consequent reduction in costs.

## Data Availability

The raw data supporting the conclusion of this article will be made available by the corresponding author without undue reservation.
